# FOR: Point Cloud Outlier Removal Based on Fuzzy Theory and Informativeness and Its Application to 3D Object Detection

**DOI:** 10.3390/s26103070

**Published:** 2026-05-13

**Authors:** Lili Gan, Zhengyi Yang, Yiyi Liu, Yaqi Wang, Xinyan An

**Affiliations:** School of Big Data and Software Engineering, Chongqing University, Chongqing 401331, China; 20191590@cqu.edu.cn (L.G.); 13626948791@163.com (Y.L.); 19923085152@163.com (Y.W.); 15280766126@163.com (X.A.)

**Keywords:** point cloud, fuzzy theory, outlier removal, denoising filtering

## Abstract

LiDAR is widely used in autonomous driving. Although LiDAR point cloud data can provide stable and reliable information about the environment, it also faces the problem of a huge amount of data. One of the reasons is that point cloud data contains a large amount of noise and outliers. Outlier removal of point clouds can reduce the impact of these disturbances and improve the quality of the point cloud, but it will inevitably eliminate some valid points, which affects subsequent perception tasks. To overcome this limitation, this paper proposes a fuzzy outlier removal (FOR) method based on fuzzy theory and informativeness. It uses fuzzy theory to model the uncertainty of the membership degree of each point in each dimension, calculates the informativeness sum of each point based on membership degree, and filters points according to the informativeness. FOR is characterized by filtering the point cloud in the edge region on the premise of retaining the point cloud in the center region, so as to preserve the environmental information in the center region and reduce the impact of outlier removal on subsequent perception tasks. The experiments focus on the contradictory relationship between outlier removal and perception accuracy, and verify the effectiveness of FOR with multiple object detection models on the autonomous driving datasets KITTI and nuScenes. The experimental results indicate that, compared with other point cloud outlier removal methods, FOR has the advantage of reducing inference time while retaining detection accuracy, demonstrating balanced high performance across different datasets and detection models.

## 1. Introduction

With the continuous advancement of LiDAR technology and cost reduction, LiDAR has been used as an advanced environment-sensing sensor and applied to many fields such as autonomous driving, aerospace, terrain survey, etc. [1,2,3]. LiDAR mainly acquires point clouds by transmitting and receiving electromagnetic waves to realize the survey of the environment. High-resolution LiDAR can obtain hundreds of thousands of point clouds in each frame, providing an adequate representation of the scene.

**Figure 1:**
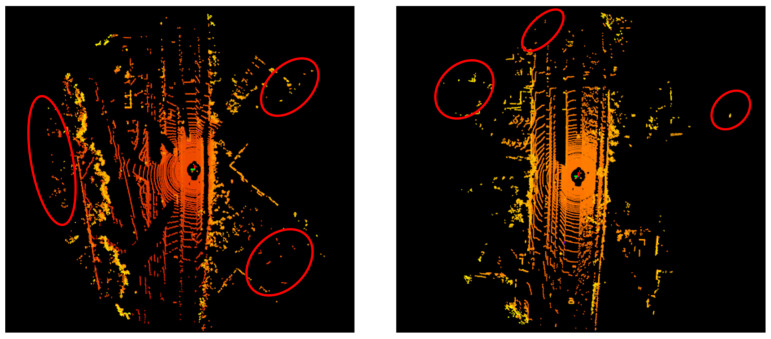
Visualization of the KITTI dataset; outliers are more distributed in the far distance.

Due to the limitations of sensor and measurement errors, point cloud data often contain a large amount of noise and large number of outliers, which not only brings great challenges for data storage and representation and computational resource consumption, but also hinders the application of downstream geometric processing [4,5]. Therefore, point cloud outlier removal has become one of the key technologies of preprocessing. The reasons for outlier generation include the physical properties of LiDAR, in addition to factors such as illumination and natural environment. As the laser of the LiDAR is emitted from the center to the surrounding 360°, the received point cloud shows an uneven density distribution. The points in the regions farther away from the center are sparser, and usually lose the geometric features of the object and become outlier points. Vehicle-mounted LiDAR can sense obstacles up to 500 m away, while the edge range contains more outliers (as shown in Figure 1). At the same time, targets closer to the center on actual traffic roads are of more concern, and existing detection techniques are more efficient for closer targets. This indicates that an outlier removal technique specialized for long-range noise is needed to clean the point cloud and improve its quality.

Three-dimensional object detection is an important branch in autonomous driving, and many LiDAR-based 3D object detection techniques have emerged in recent years and made significant breakthroughs in this direction. Unfortunately, these studies have rarely considered outlier removal in point clouds, and a few studies have only focused on point cloud outlier removal under extreme weather [6,7]. Limited by the physical properties of LiDAR, as the distance increases, the intensity of the laser signal will be attenuated due to atmospheric absorption and scattering, resulting in a weaker echo signal and a sparser point cloud in the distance. In addition, the echo signals in the distance may be subject to more noise interference, which generates more outliers in the point cloud.

There are two main reasons why existing point cloud outlier removal methods are seldom applied in 3D object detection:

(1) Limitations of local feature analysis. Existing methods such as ROR and SOR mainly rely on local features to discriminate outliers, lacking consideration of the global spatial position of points. ROR judges a point as an outlier if the number of neighbors within a fixed radius falls below a threshold; SOR treats points deviating from the local mean distance by more than a standard deviation ratio as outliers. Because these methods only analyze local geometric properties without incorporating the overall spatial distribution, they struggle to effectively remove distant and dense noise points.

(2) Binary classification defects. Traditional methods adopt binary (hard) thresholding for noise discrimination, which ignores the continuous and uncertain nature of noise intensity. This rigid decision is prone to misclassifying valid points in the center region, resulting in the loss of critical valid points and seriously degrading 3D object detection performance. This creates a fundamental contradiction between outlier removal and detection accuracy.

To address the above problems, the main contributions of this paper are as follows:

(1) A fuzzy informativeness-driven outlier removal algorithm (FOR). We propose a novel filtering method based on fuzzy theory and informativeness. The spatial position of each point is mapped to a three-dimensional fuzzy membership vector, reflecting the confidence probability of belonging to the real target surface. A weighted logarithmic informativeness model is constructed to quantify the uncertainty degree (i.e., noise likelihood) of each point. Based on global informativeness ranking, the top k% high-informativeness points are dynamically truncated, filtering distant edge-region noise while maximally preserving center-region object features.

(2) Validation on public datasets. Based on multiple advanced detection models on KITTI and nuScenes, we explore the contradictory relationship between outlier removal and detection performance. The results show that FOR achieves optimal performance on KITTI. On nuScenes, though slightly lower than NiOR and SOR, FOR still outperforms DROR and ROR, exhibiting stable cross-platform adaptability.

The rest of the paper is organized as follows: the second part reviews the related research in this paper, and the third part proposes FOR based on fuzzy theory and informativeness. Then, in the fourth part, relevant experiments are conducted to verify the effectiveness of FOR. The last part summarizes the content of this paper.

## 2. Related Work

In recent years, many studies have centered around point cloud outlier removal, from filtering-based methods to deep learning-based methods [8,9]. Among the traditional filter-based outlier removal methods, the classical methods are radius outlier removal (ROR) and statistical outlier removal (SOR). Among them, ROR considers the density within the neighborhood of a point, and if the density of the neighborhood is too small, the point is regarded as an outlier. SOR performs a statistical analysis of the neighborhood of each point and filters out some outliers that do not meet the requirements based on the features of the distribution of distances from the point to all neighboring points. Many studies have optimized these two methods and proposed new methods in research on ROR. Duan et al. [10] proposed an adaptive radius outlier removal filter based on principal component analysis (PCA), which used PCA to downsize the point cloud to get 2D point cloud data in order to reduce the time complexity of the algorithm, and then used the 2D principal components to adaptively adjust the neighborhood search radius of each point. Similarly, Makhluk et al. [11] used the Euclidean distance from the point to the coordinate origin and additional parameters to dynamize the search neighborhood of each point, and the algorithm had a good filtering effect in extreme weather. Szutor et al. [12], for traditional ROR, needed to carry out the nearest-neighbor search many times, which led to an increase in computation, combined with the idea of the cell-based filter to improve the time performance of the algorithm. In studies on SOR, Haris et al. [13] extended traditional SOR by fast clustering statistical filtering, which clustered the input point cloud, filtered the clusters with higher density by taking the average value of all clusters’ points as the threshold, and then filtered the remaining point cloud by the traditional SOR method. This method sped up the computation by being the first to retain the denser part of the point cloud through clustering and reduced the number of points and clusters actually estimated. Carrilho et al. [14] proposed an adaptive scheme based on the cellular subdivision of the point cloud. Instead of computing a single histogram for the entire dataset, the method filtered smaller patches so that ground elevation differences were ignored.

Deep learning-based point cloud outlier removal methods focus on learning and representing the features of outliers in the learning stage and identifying the points with similar features in the running stage. Rakotosaona et al. [15] proposed a method for removing outliers and reducing noise based on the structure of PCPNet [16], which utilized a network model to directly classify and discard outlier points. Pistilli et al. [17] proposed an outlier removal method based on graph convolutional neural networks, which dynamically constructed a neighborhood graph based on the similarity of high-dimensional feature representations of points, constructed a complex hierarchy of features, and utilized a convolutional layer to classify and discard the outliers. Li et al. [18] proposed a single-stage point cloud cleaning network (SSPCN) for implementing outlier filtration and denoising of point clouds, in which an improved density-based farthest point sampling (DBFPS) method was used for downsampling and outlier removal.

Deep learning-based outlier removal methods need to represent the high-dimensional features of the point cloud, ignoring the basic location information of point clouds, so most studies still focus on filtering-based outlier removal algorithms [19,20,21,22]. In addition, there are some novel outlier removal methods. For example, Li et al. [23] proposed PointCVaR, which defined the concept of point risk and reshaped the outlier removal process into an optimization problem using gradient-based attribution analysis.

Existing research methods do not focus on the removal of outliers from the point cloud, and fail to find a balance between detection time and detection accuracy, always paying too much attention to one side and ignoring the other. The FOR proposed in this paper will maximize the retention of point cloud features in the central region of the scene under the premise of filtering the distant noise and outliers and ensure detection accuracy while reducing the detection time and improving the detection performance.

## 3. Method

Aiming to address the limitations of traditional filtering-based point cloud outlier removal methods in 3D object detection, we propose FOR based on fuzzy theory and informativeness to filter the point cloud from a distance. Since distance is a precise quantitative metric, but ’near’ and ’far’ are fuzzy concepts in reality, fuzzy theory is used to model the uncertainty of the membership of points in the central region in order to better portray these fuzzy concepts.

### 3.1. Fuzzy Theory and Informativeness

**Fuzzy theory.** Fuzzy set theory is a tool used to represent uncertainty and ambiguity, i.e., to express the assessment of things in terms of numbers between 0 and 1. In recent years, the concept of fuzzy set theory has been gradually introduced into point cloud data processing and 3D object detection [24,25,26]. Fuzzy set theory is defined as follows:

For a given mapping over the discussion domain, as in Equation (1),

(1) $$\mu_{A}:X\to \left[0,1\right]$$

where A is a fuzzy subset on the discussion domain X, $\mu_{A}()$ is the membership function of A, and $\mu_{A}(x)$ is the degree to which x is attributed to A, expressed as in Equation (2):

(2) $$A=\{\left(x,\mu_{A}\left(x\right)\right)|\forall x\in X\}$$

As in a point cloud, the magnitude of the degree of membership of a point P on the x-axis to the entire point cloud data is indicated $\mu_{X}(P)$. A common way to find the degree of membership is to simplify the membership function into a triangle. Let A be a subset of fuzzy numbers on the set of real numbers R. Its membership function $\mu_{A}$ is a continuous mapping from the real number domain to the interval [0, 1].

The membership function of a triangular fuzzy set is calculated as follows:

(3) $$\mu_{A}\left(x\right)=\{\begin{array}{c} (x-c+\delta )/(a-c+\delta ),c\leq x\leq a\\ (x-b-\delta )/(a-b-\delta ),a\leq x\leq b. \end{array}$$

where c and b denote the lower and upper limits of the assessment variables, respectively, a denotes the intermediate value, and the average of c and b is usually taken. As x gets closer to a, $\mu_{A}(x)$ gets closer to 1, indicating higher membership of x to A. Conversely, the closer $\mu_{A}(x)$ is to 0, the lower the membership of x to A. δ is usually used to adjust the range of $\mu_{A}(x)$ so that it is in (0, 1]. In the point cloud data, the minimum value of c, the maximum value of b, and the average value of a are found for all the point clouds on the x-axis. For the value of the x-axis of each point cloud, the closer the points are to c and b, the more likely they are to be edge points and noisy points, and the closer the points are to a, the more likely they are to be points near the center. The same is true for the y-axis and the z-axis. In this paper, δ is set to δ = (b − c)/n, where n denotes the number of evaluation variables, i.e., the number of points in the space.

For the probability of a single event, the uncertainty can be measured in terms of the informativeness, as in Equation (4). Informativeness H is a concept based on informativeness theory and can be intuitively interpreted as the ‘degree of unexpectedness’ or ‘uncertainty’. Compared with the degree of membership, the scale is unified, which can be better analyzed.

(4) H=−logp

It is easy to prove that the function curve of Equation (4) is monotonically decreasing in the interval (0, 1], i.e., the higher the probability for a single event, the less informative it is and the less uncertain it is. Conversely, the smaller the probability, the more informative and the greater the uncertainty. Therefore, informativeness can be used to measure the uncertainty of the proposition of whether the point cloud belongs to the center region. Frankly speaking, informativeness H(p) = −log(p) measures the ’unexpected degree’ of the point cloud location: the informativeness of the central region point (high p) is low, because its location is expected to be clear; edge points (low p) contain a lot of informativeness, which may be outliers or invalid data. Unlike the probability estimation, the location of the point cloud is certain in space, and the probability is expressed by the membership degree of the point cloud with the center region, which is also certain, and the uncertainty of this proposition should be as small as possible.

In consideration of the additivity of the informativeness, the sum of the informativeness of each point can be computed and weighted according to its membership degree on the x, y, and z axes, as in Equation (5).

(5) $$E_{i}=-\alpha log\mu_{X}\left(P_{i}\right)-\beta log\mu_{Y}\left(P_{i}\right)-\gamma log\mu_{Z}\left(P_{i}\right)$$

where $\mu_{j}\left(P_{i}\right)$, j∈{X,Y,Z} denotes the membership of the ith point to each coordinate axis and α, β, γ denote the weight of each dimension, respectively. FOR aims to filter the outlier points at a distance, so focusing on the distribution of the point cloud in the XOY plane, the computation and experiments in this paper set the weights as α = 0.4, β = 0.4, γ = 0.2.

**Figure 2:**
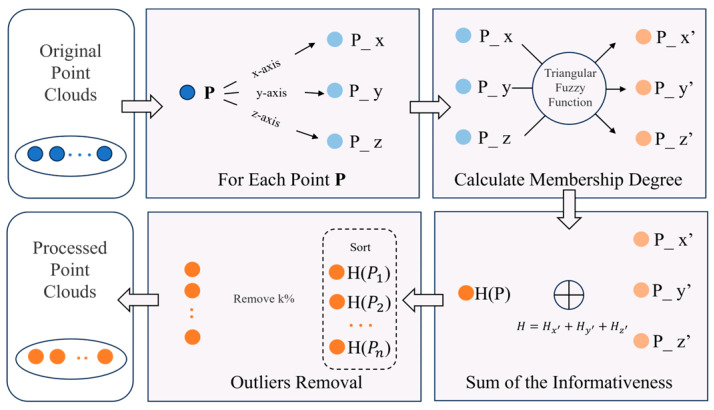
FOR framework.

FOR combines fuzzy theory and informativeness metrics, skillfully using membership degree to model the uncertainty of point clouds and screen out the most likely point clouds in the central region. The overall framework of FOR is shown in Figure 2.

### 3.2. FOR Algorithm

FOR calculates the membership of each point cloud on different axes with respect to the center region. It considers the membership as a probability and calculates the sum of the amount of informativeness of each point cloud on each axis. The membership obtained by the points in the edge region is low, and the sum of the informativeness is large. After calculating the sum of the informativeness of each point, the points are sorted by the size of informativeness. According to the set outlier ratio k (e.g., 0.2), which indicates that 20% of the points will be removed, the points with the top 20% of the total informativeness will be filtered out. The specific steps are shown in Algorithm 1.
**Algorithm 1 FOR****Input**: Points                         - A set of point clouds to be filtered k                         - Outlier ratio **Output**: denosiedPoints                     - Point cloud after outlier removal # Calculate fuzzy number 1     fuzzy_num = [min(points, axis = 0),0, max(points, axis = 0)] 2     entropy_list = [] 3     **for** p∈points **do** # Calculate the membership of each point 4       membership = Membership(fuzzy_num, p) # Calculating informativeness 5       e = CalculateInformativeness(membership) 6       informativeness_list.append(e) 7     **end for** 8     Sort(informativeness_list) # Find the informativeness threshold 9     threshold = Search(informativeness_list, k) 10  **for all** i **do** 11     **if** informativeness_list [i] > threshold: 12         denosiedPoints = Filter(points, points[i]) 13     **end if** 14  **end for** 15  **return** denosiedPoints

In order to illustrate the flow of the algorithm more intuitively, the details of the algorithm are demonstrated through an arithmetic example.

**Step 1:** Let the space include 10,000 points, and construct the triangular fuzzy number of the x, y, and z axes based on the coordinate values of all point clouds. As:

x_- min = −77.28; x_- mid = 0; x_- max = 78.69;

y_- min = −67.55; y_- mid = 0; y_- max = 55.48;

z_- min = −9.56; z_- mid = 0; z_- max = 2.78.

**Step 2:** Calculate the degree of membership of each point on each axis using Equation (2), e.g., the coordinates of a point P are (−17.5, 18, −0.9). Then, its degree of membership on each of the three coordinate axes is μ_X (P) = 0.773, μ_Y (P) = 0.672, μ_Z (P) = 0.905.

**Step 3:** Weigh the sum of the informativeness of the point according to Equation (5) to obtain E_P = 0.122.

**Step 4:** Find the informativeness of each point and sort it by size. According to the set proportion of the outlier value, remove the points outside the range.

**Figure 3:**
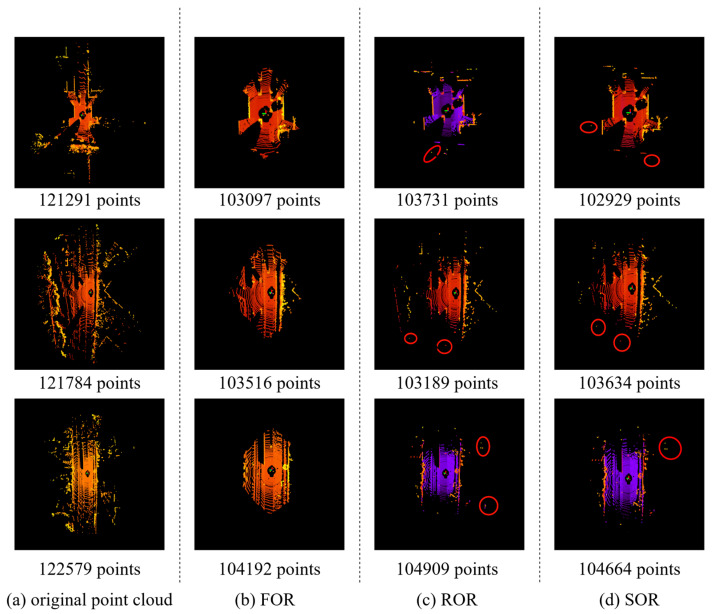
Visualization of filtering results.

The actual filtering effect of FOR is shown in Figure 3 and compared with the other two commonly used outlier removal algorithms through the parameters of each algorithm, so that the difference between the number of points they retained is within 1000. In this case, the effect of FOR is similar to that of the other methods, which verifies the validity of FOR and the two outlier removal methods, SOR and ROR. There are still some noise points in the range of the edges that cannot be filtered. At the same time, FOR focuses more on retaining the point cloud in the center region, so the distribution of the filtered point cloud is more concentrated. Compared with other methods, it can be adjusted by fewer parameters. The effect of FOR on the object detection results and its performance analysis will be discussed in detail in Section 4, Experiment.

### 3.3. Time Complexity

The time complexity statistics of FOR are shown in Table 1. The time complexity of FOR mainly focuses on the step of sorting the informativeness for each point, so its time complexity is N*(logN). SOR and ROR, on the other hand, mainly traverse each point and search for the neighboring points of each point, in which the search process is realized by using a spatial partitioning search structure on the basis of kd-tree, with a complexity of O(logN) and an overall time complexity of O(N*logN). Thus, FOR has the same time complexity as SOR and ROR.

## 4. Experiment

### 4.1. Experimental Setup

In order to evaluate the effectiveness of FOR, validation is performed on the autonomous driving open-source dataset using several 3D object detection models. The experiment is conducted on the validation sets of the KITTI [27] and nuScenes [28] datasets, with the main objective of exploring the accuracy loss of the data after FOR under different detection models. In terms of data sample distribution, the validation set of the KITTI dataset (64-line LiDAR) contains 3769 frames of point cloud data, which mainly detects the three categories of cars, pedestrians, and cyclists. The validation set of the nuScenes dataset (32-line LiDAR) contains 150 scenes of continuous point cloud data, which mainly detects 10 categories such as cars, pedestrians, and trucks. In terms of evaluation metrics, the KITTI dataset is evaluated in four aspects: 3D bounding box, 2D bounding box, bird’s-eye view (BEV), and average orientation similarity (AOS).

**Three-dimensional**: The accuracy of the 3D bounding box of an object is predicted using the IOU as a reference standard, and if the IOU is greater than a set threshold, the prediction is a positive sample.

**Two-dimensional**: The predicted frame is projected to the camera view through the camera parameters, and its accuracy is calculated in the 2D plane, again judging positive and negative samples based on the IOU.

**BEV**: The prediction frame is projected to the bird’s-eye view perspective to calculate its accuracy, and the positive and negative samples are judged according to the IOU.

**AOS**: This is an accuracy index that indicates the orientation of the prediction frame. Unlike 2D detection, 3D object detection usually needs to pay attention to the orientation information of the target. The AOS is expressed by the cosine similarity (OS) of the two direction vectors of the angle of the prediction frame (θ_pred) and the angle of the real frame (θ_gt), as in Equation (6), and then the OS of each category is averaged to obtain the average cosine similarity (AOS).

(6) $$OS=\left|cos\left(\theta_{pred}-\theta_{gt}\right)\right|$$

In addition, two metrics, AP11 and AP40, can be considered depending on the different settings of the accuracy thresholds. The nuScenes dataset defines an error metric mTP = {mATE, mASE, mAOE, mAVE, mAAE}, in addition to the average accuracy of detecting the bounding box, and a comprehensive evaluation metric, NDS, the details of which can be viewed in [28].

In order to get more comprehensive evaluation results, the selected detection models should all differ in terms of algorithms, which are MVF [29], MVXNet [30], Part-A^2^ [31], and PointPillars [32]. Among them, MVF employs a multi-view fusion detection method, MVXNet is a classic in multi-modal fusion detection schemes, and Part-A^2^ introduces partial perception and aggregation. PointPillars, on the other hand, employs a pillar-based encoding of point cloud features. All detectors (MVF, MVXNet, Part-A^2^, PointPillars) use publicly available pre-trained weights provided by the original authors or the MMDetection3D framework. Table 2 shows the same configuration used in the experiment.

### 4.2. Experimental Results

In addition to the classical ROR and SOR, the Dynamic Radius Outlier Removal Filter (DROR) [7] proposed by Charron et al. and the Non-isolated outlier removal (NiOR) proposed by Ning et al. [33] are also referenced. Specifically, the parameters of each filtering method are adjusted so that the number of retained points differs by less than 1000 points per frame compared to FOR-0.25 (which retains approximately 75% of original points). This principle is applied consistently in both the qualitative visualization (Figure 3) and all quantitative comparisons on KITTI and nuScenes.

**Figure 4:**
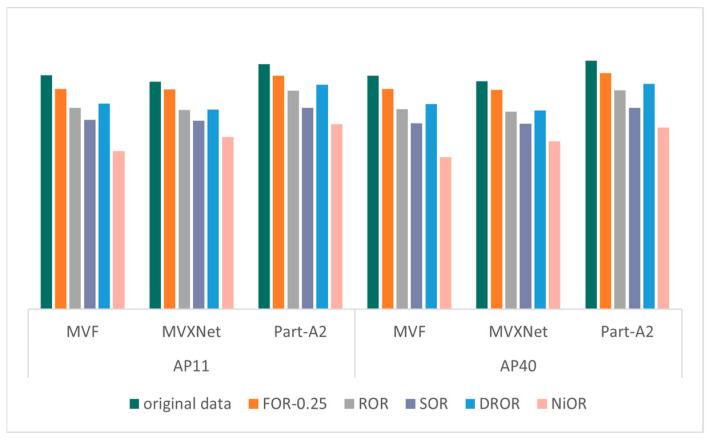
The mAP of 3D bounding box.

**Figure 5:**
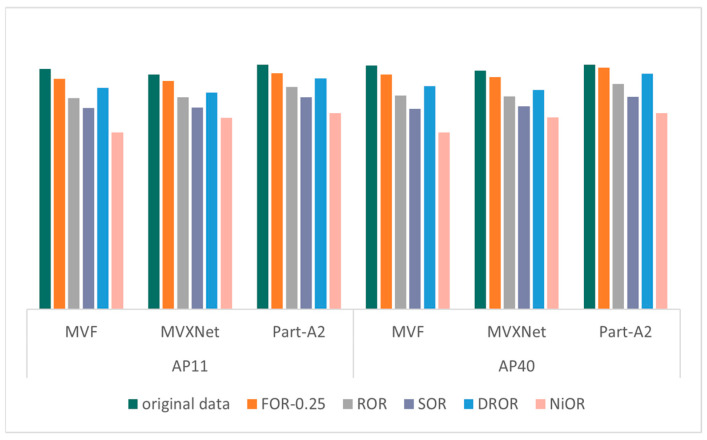
The mAP of 2D bounding box.

**Figure 6:**
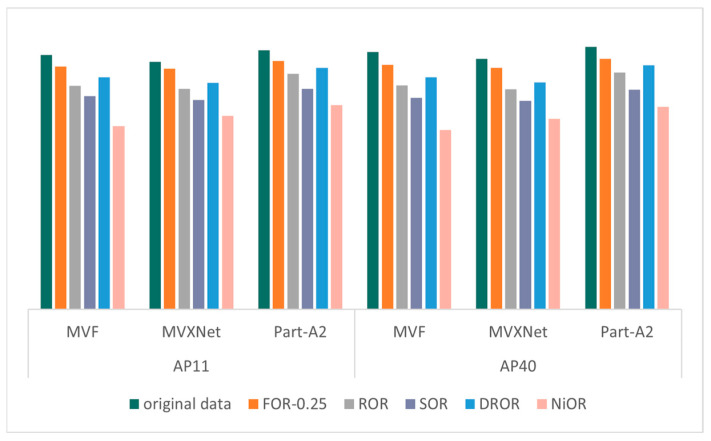
The mAP of BEV.

**Figure 7:**
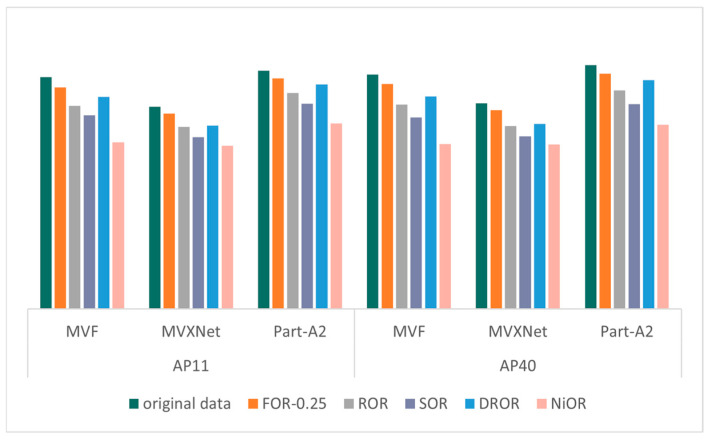
The mAP of AOS.

The validation results of the KITTI dataset on different models after different outlier removal methods are shown in Figure 4, Figure 5, Figure 6 and Figure 7, which record the precision of the three models under four metrics: 2D bounding box, 3D bounding box, BEV bounding box, and AOS, where the precision values are the average of the precision of the three categories (cars, pedestrians, and cyclists) and are partitioned according to the recall points (AP11 and AP40). The specific mAP values in Figure 4, Figure 5, Figure 6 and Figure 7 are shown in Table A1, Table A2, Table A3 and Table A4 in Appendix A. The outlier ratio for FOR (orange in Figure 4, Figure 5, Figure 6 and Figure 7) is set to 0.25, indicating that 25% of the points in each frame of data are filtered out. The ROR (gray in Figure 4, Figure 5, Figure 6 and Figure 7) parameter uses the default parameters radius = 0.5 and number of neighborhood points = 10. The default parameters for SOR (purple in Figure 4, Figure 5, Figure 6 and Figure 7) are standard deviation ratio = 0.5 and number of neighborhood points = 10. For DROR (blue in Figure 4, Figure 5, Figure 6 and Figure 7), the number of neighborhood points is 10 and the minimum radius is 0.5. For NiOR (pink in Figure 4, Figure 5, Figure 6 and Figure 7), the number of neighborhood is 10 and the threshold is 0.55. By analyzing Figure 4, Figure 5, Figure 6 and Figure 7, it can be concluded that the accuracy of each outlier removal method on different models is, in descending order, FOR > DROR > ROR > SOR > NiOR.

After filtering some points from the original data, which include some points of objects in addition to noise, the accuracy of the filtered data is all reduced compared to the original data. However, in each metric, the accuracy of FOR is closest to the accuracy of the original data, and the error in the accuracy of each metric compared to the original data is around 5%, which is much smaller than the error of the other four outlier removal methods. This is because FOR gives priority to ensuring the point cloud within the center neighborhood from the overall point cloud density, and the center region is the region with the highest point cloud density and the most obvious object features. Therefore, FOR effectively protects the features within this region, thus minimizing the object detection error. Other outlier removal methods filter from local density without considering the problem of excessive density in the center region, which leads to the loss of more effective points, and, thus, the accuracy is seriously degraded.

Table 3 shows the validation results of Pointpillars on the nuScenes-trainval dataset after processing with different outlier removal methods, and according to the comprehensive evaluation metric NDS, the comprehensive performance of each outlier removal method, in descending order, is as follows: NiOR> SOR> FOR> ROR> DROR. Combined with their performance on KITTI, NiOR and SOR perform well on nuScenes but decline significantly on KITTI; DROR and ROR show the opposite trend. In contrast, FOR achieves optimal performance on KITTI. On nuScenes, though slightly lower than NiOR and SOR, FOR still outperforms DROR and ROR, exhibiting no significant performance fluctuations across different sensor configurations (64-line/32-line LiDAR), demonstrating good cross-platform adaptability and robustness.

Table 4 shows the FPS values of different data under different models, and it can be seen that the FPS after different outlier removal is higher than the original FPS because the filtering reduces the number of original point clouds. FOR achieves the highest FPS on KITTI across all models, with a 124% improvement on Part-A^2^. On nuScenes, FOR is sub-optimal, second only to DROR.

Combining accuracy (Table 3) and speed (Table 4) results reveals distinct method characteristics. DROR achieves the fastest inference but suffers from the lowest NDS, indicating an aggressive outlier removal strategy that sacrifices accuracy for speed. Conversely, NiOR achieves the highest NDS but shows limited FPS improvement, suggesting conservative outlier removal that preserves accuracy at the cost of efficiency. FOR occupies an optimal balance: on KITTI, it dominates both metrics (highest accuracy and highest FPS); on nuScenes, its FPS is competitive (second only to DROR), while its NDS remains close to the best performers (NiOR). This demonstrates FOR’s superior ability to simultaneously optimize accuracy and efficiency, avoiding the single-metric extremes of other methods.

Table 5 shows the detection results of FOR with different outlier ratio settings. From the table, we can analyze that on each index of each model, the detection accuracy decreases with an increase in the outlier ratio. This is due to the fact that with an increase in the outlier ratio, more points are filtered, which results in affecting the accuracy of the detection. However, the accuracies at different outlier ratios are very close to each other, and the error between each noise point ratio and the neighboring noise point ratios is within 1%, which indicates that the parameter settings of FOR have a small impact on the detection. According to the statistics, the average effective area of each frame in the KITTI dataset consists of 18,560 points, and the difference between different noise point ratios is 5%, which means that each time we adjust the parameters, we will filter out about 928 more points, and these 928 points are filtered from the farthest range, which will not affect the objects in the center area and minimize the loss of accuracy.

## 5. Conclusions

As a fundamental environment perception sensor, LiDAR generates precise 3D representations of real-world scenes through point cloud data, with widespread applications in autonomous driving and related fields. However, conventional outlier removal processes inevitably eliminate valid points during noise suppression, adversely affecting subsequent 3D object detection performance. To resolve this critical issue, we have developed a Fuzzy Outlier Removal (FOR) method that innovatively combines fuzzy set theory with information-theoretic metrics. The method calculates the informativeness of each point by constructing the membership of each point to the point cloud space in each dimension, and filters the points according to the order of the informativeness magnitude and the proportion of outlier points. FOR filters out a large amount of noise and outliers when filtering the point cloud to reduce detection time and improve efficiency, and retains the point cloud in the center region to reduce the loss of valid points and improve accuracy, which balances the contradiction between efficiency and accuracy.

The experimental results indicate that the performance of FOR is stable across different datasets and detection models. On the KITTI dataset, FOR achieves a competitive performance in reducing inference time while minimizing detection accuracy loss compared to other outlier removal methods. On the nuScenes dataset, FOR demonstrates a stable performance, though specific methods may excel in particular metrics (e.g., NiOR achieves higher NDS, while DROR achieves higher FPS). These mixed results suggest that FOR’s strength lies in its consistent ability to balance accuracy and efficiency, particularly in center-region preservation, rather than dominating all metrics across all datasets. Future work will focus on developing specific response mechanisms for local density variations, as the current method only considers the global position of points.

## Figures and Tables

**Table 1:** Time complexity statistics of FOR.

Step	Operation	Time Complexity
Calculate boundary values for each axis	Iterate over each coordinate value of each point and compute the boundary value	3*O(N)
Calculate the membership of each point	Iterate over each point	O(N)
Calculate informativeness at each point	Iterate over each point	3*O(N)
Sorting informativeness	Using the Quick Sort Algorithm	O(N*logN)
Filtering point clouds	Iterate over the informativeness at each point	O(N)

**Table 2:** Configuration of Experimental Environment.

Experimental Environment	Configuration/Version Description
CPU	Intel Core (TM) i9-12900k, 24 cores
GPU	NVIDIA GeForce RTX 3090, 24 GB
Programming Language	Python 3.8.15
Deep Learning Framework	Pytorch 1.9.0, CUDA 11.1

**Table 3:** Pointpillars in the nuScenes-trainval dataset.

Assessment of Indicators	mAP	mATE	mASE	mAOE	mAVE	mAEE	NDS
original data	0.2918	0.4698	0.3504	1.5430	0.3752	0.1912	**0.4072**
ROR	0.2607	0.4890	0.3555	1.5462	0.3792	0.2025	**0.3877**
SOR	0.2726	0.4796	0.3530	1.5471	0.3642	0.1966	**0.3970**
DROR	0.2562	0.4959	0.3573	1.5480	0.3992	0.2038	**0.3825**
NiOR	0.2820	0.4758	0.3520	1.5464	0.3740	0.1946	**0.4014**
FOR-0.25	0.2720	0.4799	0.3521	1.5470	0.3702	0.1965	**0.3961**

**Table 4:** FPS comparison of different models on different datasets.

Model (Dataset)	MVF (KITTI)	MVXNet (KITTI)	Part-A^2^ (KITTI)	Pointpillars (nuScenes)
original data	24.15	11.04	8.09	21.16
FOR-0.25	**33.82**	**14.93**	**18.15**	27.00
ROR	23.57	12.09	8.81	23.23
SOR	24.33	11.81	8.61	21.73
DROR	23.24	12.33	8.55	**34.18**
NiOR	25.13	12.54	9.03	22.35

**Table 5:** Parameter sensitivity comparison of FOR.

Norm	Threshold	MVF	MVXNet	Part-A^2^
3D	FOR-0.1	66.34	64.71	69.94
	FOR-0.15	65.60	64.45	69.21
	FOR-0.2	64.63	63.57	68.45
2D	FOR-0.1	77.05	75.41	78.94
	FOR-0.15	76.67	75.10	78.51
	FOR-0.2	75.75	74.73	77.93
BEV	FOR-0.1	72.53	70.88	73.94
	FOR-0.15	71.60	70.56	73.59
	FOR-0.2	70.92	69.64	72.64
AOS	FOR-0.1	74.08	64.99	76.74
	FOR-0.15	73.72	64.48	76.33
	FOR-0.2	72.81	63.52	75.79

## Data Availability

The KITTI dataset is publicly available at http://www.cvlibs.net/datasets/kitti/ (accessed on 29 April 2026), and the nuScenes dataset is publicly available at https://www.nuscenes.org/ (accessed on 29 April 2026). Both datasets are widely used benchmarks in the autonomous driving community. The implementation code of FOR and the experimental configurations will be made available from the corresponding author upon reasonable request. The data that support the findings of this study are available from the corresponding author upon reasonable request.

## Appendix A

The specific mAP values of Figure 4, Figure 5, Figure 6 and Figure 7 in the text are shown in Appendix A.

**Table A1:** The mAP% values of 3D bounding box (Figure 4).

Methold	AP11			AP40		
	MVF	MVXNet	Part-A^2^	MVF	MVXNet	Part-A^2^
original data	66.94	65.07	70.09	66.77	65.16	71.12
FOR-0.25	63.02	62.82	66.73	63.04	62.78	67.48
ROR	57.54	56.92	62.44	57.25	56.49	62.60
SOR	54.11	53.92	57.55	53.15	53.05	57.62
DROR	58.80	57.07	64.26	58.63	56.86	64.45
NiOR	45.20	49.25	52.91	43.45	47.97	51.93

**Table A2:** The mAP% values of 2D bounding box (Figure 5).

Methold	AP11			AP40		
	MVF	MVXNet	Part-A^2^	MVF	MVXNet	Part-A^2^
original data	77.28	75.56	78.65	78.45	76.84	78.65
FOR-0.25	74.10	73.41	75.95	75.56	74.65	77.72
ROR	67.94	68.17	71.48	68.77	68.44	72.47
SOR	64.83	64.86	68.22	64.45	65.26	68.32
DROR	71.23	69.76	74.26	71.83	70.62	75.79
NiOR	56.95	61.56	63.15	56.89	61.70	63.16

**Table A3:** The mAP% values of BEV (Figure 6).

Methold	AP11			AP40		
	MVF	MVXNet	Part-A^2^	MVF	MVXNet	Part-A^2^
original data	72.74	70.84	74.14	73.60	71.71	75.12
FOR-0.25	69.53	68.89	71.06	69.96	69.15	71.71
ROR	63.92	63.10	67.43	64.04	63.00	67.77
SOR	60.96	59.91	63.06	60.48	59.63	62.87
DROR	66.43	64.86	69.08	66.40	64.91	69.88
NiOR	52.43	55.38	58.40	51.30	54.47	57.93

**Table A4:** The mAP% values of AOS (Figure 7).

Methold	AP11			AP40		
	MVF	MVXNet	Part-A^2^	MVF	MVXNet	Part-A^2^
original data	74.53	64.99	76.59	75.35	66.11	78.46
FOR-0.25	71.26	62.90	74.10	72.35	63.92	75.62
ROR	65.37	58.58	69.51	65.80	58.77	70.23
SOR	62.34	55.26	66.06	61.64	55.49	65.87
DROR	68.19	58.91	72.28	68.38	59.57	73.55
NiOR	53.60	52.51	59.64	53.04	52.86	59.19
